# Rice Lesion Mimic Gene Cloning and Association Analysis for Disease Resistance

**DOI:** 10.3390/cimb44050160

**Published:** 2022-05-22

**Authors:** Anpeng Zhang, Hongzhen Jiang, Huangwei Chu, Liming Cao, Jingguang Chen

**Affiliations:** 1 Institute of Crop Breeding and Cultivation, Shanghai Academy of Agricultural Sciences, Shanghai 201403, China; anpeng0228@163.com (A.Z.); chuhuangwei@saas.sh.cn (H.C.); 2 School of Agriculture, Shenzhen Campus of Sun Yat-sen University, Shenzhen 518107, China; jianghzh23@mail.sysu.edu.cn

**Keywords:** breeding, disease resistance mechanism, lesion mimic mutants, rice

## Abstract

Lesion mimic mutants refer to a class of mutants that naturally form necrotic lesions similar to allergic reactions on leaves in the absence of significant stress or damage and without being harmed by pathogens. Mutations in most lesion mimic genes, such as *OsACL-A2* and *OsSCYL2*, can enhance mutants’ resistance to pathogens. Lesion mimic mutants are ideal materials for studying programmed cell death (PCD) and plant defense mechanisms. Studying the genes responsible for the rice disease-like phenotype is of great significance for understanding the disease resistance mechanism of rice. In this paper, the nomenclature, occurrence mechanism, genetic characteristics, regulatory pathways, and the research progress on the cloning and disease resistance of rice lesion mimic mutant genes were reviewed, in order to further analyze the various lesion mimic mutants of rice. The mechanism lays a theoretical foundation and provides a reference for rice breeding.

## 1. Introduction

Rice is one of the most important food crops in the world and plays an important role in people’s lives. Diseases are one of the important factors affecting rice production. Rice production is often threatened by various diseases such as rice blast, bacterial blight, and rice false smut. When the disease is severe, it will cause a substantial reduction in rice production [1]. The use of chemical drugs to prevent and control diseases not only increases the resistance of diseases to drugs, but also seriously pollutes the environment. At present, the most effective and least harmful way to prevent diseases is to cultivate rice varieties with excellent resistance.

To adapt to various biotic and abiotic stresses in the natural environment, plants have evolved a complex and effective defense system by adjusting their metabolism and structure to cope with external unfavorable factors. Hypersensitive response (HR) is one of the most effective methods for plants to fight pathogens [2]. After being infected by non-affinity pathogens, the plant body produces an allergic reaction, and the cells in the infected part die rapidly, thereby limiting the expansion of the pathogenic bacteria to the uninfected part, which is a kind of programmed cell death process [3]. PCD is a gene-regulated “active suicide”, which is an important biological process for eukaryotes to remove their own damaged or redundant cells in a targeted manner. Plant PCD is involved in stress response and growth and development [4]. Plant HR is caused by the interaction between resistance proteins and avirulent genes of pathogenic bacteria, which is usually accompanied by related ROS burst, cell wall thickening, pathogen-related gene expression, and accumulation of antibacterial substances [5].

Lesion mimic mutants refer to a class of mutants that have not been infected by pathogens but have typical necrotic lesions [6]. Not only in rice, disease-like spots often occur in a variety of plants such as corn, barley, and Arabidopsis [7]. The appearance of disease-mimicking spots is usually accompanied by programmed cell death (PCD), and stimulates the plant’s self-defense response, induces high expression of defense genes, and enhances plant resistance to multiple pathogens such as rice blast and bacterial blight. Therefore, lesion mimic mutants are natural materials for studying the mechanisms of plant programmed death and defense response.

## 2. Characteristics and Nomenclature of Lesion Mimic Mutants

The Japanese scientist Sekiguchi reported the first disease-like mutant produced by natural mutation of rice, and this type of disease-like mutant was named *sekiguchi lesion* [8]. At present, for rice lesion mimic mutants, researchers have roughly named them in the following two ways: one is the nomenclature that refers to the international standard for genetic research on cereal crops with the prefix lesion resembling disease (lrd); the other is spotted leaf (spl), brown leaf spot (bl), cell death and resistance (cdr), lesion mimic resembling (lmr), and lesion mimic mutant (lmm), prefixed with mutant phenotype or physiological characteristics, and spl most often observed as a prefix [9,10].

Spot-like mutant is an extremely broad concept, so its phenotype also presents a variety of characteristics, but it mainly appears in leaves and leaf sheaths, particularly in leaves, and very few appear on ears. Correspondingly, the color of the lesion mimic phenotype is diverse, mainly dark and light brown, including a few orange-yellow (e.g., *spl1*) and white lesions (e.g., *spl20*) [10,11]. Since descriptions of lesion phenotypes are mostly visual observations, there may be some differences in this description for different observers. To this end, Wu et al. [7] obtained a generalization of the phenotype of rice disease-like spots by analyzing 21 rice lesion mimic mutants; with respect to the color of the disease-like phenotype, brown spots were the primary ones. But the shades are not the same, and there are color phenotypes such as gray and red in addition to orange and white. From the outline shape of the lesion mimic phenotype, circle, ellipse and rod are the primary ones, and there are some different regular contour shapes; the size and distribution of the spots are different and vary greatly, some spots are large and sparse, while some are small and denser. With respect to appearance time, the disease-like phenotype mainly appeared at the seedling stage, including the phenotype that appeared only at the tillering stage, and some of the disease-like spots did not grow or disappear as the plant grew to the mature stage. In addition to this, abiotic factors such as temperature and light also affect the appearance of phenotypes. The disease-like mutant *spl7* of rice is significantly affected by temperature. Under the condition of high temperature (30–35 °C), the disease-like phenotype will appear in large numbers, whereas when under low temperature (26 °C), the phenotype will be significantly reduced [12]. In the lesion mimic mutant of *spl5*, the researchers found no lesion phenotype in the shaded part after shading the leaves [13].

According to the period of appearance and development of the lesion mimic phenotype, Walbot et al. [14] divided it into two types: initial type and diffuse type. In a report, the diffuse type is also called feedback type. The so-called initiation type is a lesion mimic phenotype induced by PCD, which exhibits stable spot size and outline and does not spread further along with growth like *blm* reported in rice [15]; The other is the opposite, because the accumulation of reactive oxygen species is inhibited. When the lesion mimic phenotype appears, it will spread in most cases and even lead to complete leaf dieback, such as *spl1* reported in rice [11].

## 3. The Pathogenesis of Plant Lesion Mimic Mutants

**Figure 1:**
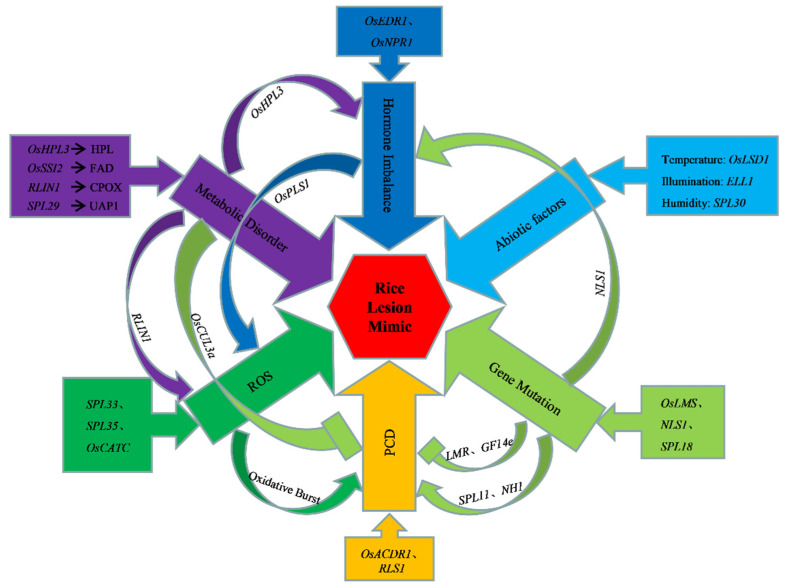
Mechanism of some rice lesion mimic mutations. PCD: Programmed Cell Death; ROS: Reactive Oxygen Species; HPL, Hydroperoxide Lyase; FAD, Fatty Acid Desaturase; UAP1, Uridinedipho Acetylglucosamine Pyrophosphorylase 1.

With the in-depth study of the disease-like plaques, the pathogenesis of the disease-like plaques has been continuously discovered, including gene mutation, hormone imbalance, metabolic disorder, disorder of PCD, accumulation of reactive oxygen species, abiotic stress, etc. (Figure 1).

### 3.1. Gene Mutation

When the gene expression is abnormal, the disease-like phenomenon will occur. Abnormal expression or mutation of disease resistance genes will lead to abnormal expression of defense-related genes, which will lead to disturbance of defense response signaling pathways, and eventually lead to plant cell death and disease-like spots. For example, after the *NLS1* of the CC-NB-LRR protein encoded by rice is mutated, H_2_O_2_ and salicylic acid (SA) will accumulate in the mutants, resulting in high expression of resistance-related genes. Abnormal expression eventually led to disease-like spots in rice leaf sheaths [16]. 

### 3.2. Hormone Imbalance

Plant hormones refer to a class of small molecular organic compounds that exist in plants and can regulate plant growth and development, mainly including auxin, cytokinin, gibberellin, ethylene, salicylic acid and jasmonic acid. Among them, ethylene, salicylic acid, and jasmonic acid, as important signaling molecules in plants, play various roles in defense responses. Lesion mimic plaques can also occur when ethylene, salicylic acid, and jasmonic acid are disrupted in their transmission as hormone signaling molecules. Jiang et al. [17] found that the increase in the level of endogenous salicylic acid in the rice transposon insertion mutant *ssil2* induced a significant increase in the expression of WRKY45, a key factor regulating rice resistance, resulting in a rice disease-like mutation. Shen et al. [18] knocked out the *OsEDR1* gene in rice plants, resulting in increased levels of salicylic acid and jasmonic acid hormones and decreased levels of ethylene hormones in the resulting plants, resulting in the generation of rice lesion mimic mutants.

### 3.3. Metabolic Disorder

Lesion mimic plaques can also occur when proteases or signaling molecules are abnormal. Plant growth and metabolism are jointly regulated by a variety of enzymes and proteins. The activity of these enzymes or proteins is reduced or their functions are lost, causing plant metabolic disorder, which in turn leads to the production of disease-like spots. Rice *OsHPL3* and *OsSSI2* genes encode lipid hydroperoxide lyase and fatty acid dehydrogenase in the fatty acid metabolism pathway, respectively. The loss of *OsHPL3* and *OsSSI2* gene function can lead to the disease-like phenotype of rice leaves [17,19]. Mutation of the rice *RLIN1* gene can alter the activity of coproporphyrinogen III oxidase, resulting in the production of necrotic spots in rice leaves [20]. Sakuraba et al. [21] reported that the rice lesion mimic mutant *fgl* prematurely terminated the synthesis of protochlorophyllate oxidoreductase B in advance due to the frameshift mutation in the second exon of the gene related to the coding of protochlorophyllate oxidoreductase, resulting in the disorder of chlorophyll metabolism and the emergence of plaque-like disease in the rice leaves.

### 3.4. Disorder of PCD

Abnormal PCD process in rice can also cause disease-like spots in rice. In order to adapt to adverse conditions in the environment, plants have developed a complex and effective mechanism for regulating cell death during evolution. PCD is an orderly and active death process regulated by a certain gene in organisms. Any factor that causes the normal PCD disorder in plants may induce the disease-like phenotype. The rice *OsACDR1* gene encodes raf-like mitogen-activated protein kinases (MAPKs), which promote cell death in plants. Overexpression of this gene also induces a lesion mimic phenotype and leads to up-regulated expression of defense response-related genes, and phenolic compounds and secondary metabolite accumulation, in addition to increased resistance to rice blast [22]. The rice gene *GF14e* encodes a 14-3-3 protein, which is a negative regulator of cell death and defense responses in rice, and interferes with plants exhibiting a spot-like phenotype and enhanced resistance to *M. oryzae* and bacterial blight [23]. Zeng et al. presented that the rice *SPL11* gene negatively regulates rice defense response and cell death process by encoding U-Box/ARM protein with E3 ubiquitin ligase function [24]. Liu et al. found that *OsCUL3a* mutation produces a lesion mimic phenotype, and OsCUL3a interacts with OsNPR1 to target degradation to mediate 26S proteasomal degradation, thereby negatively regulating the PCD process in rice [25].

### 3.5. Accumulation of Reactive Oxygen Species

The occurrence of many lesion-mimicking mutants is closely related to the accumulation of reactive oxygen species in plants. Lin et al. found that the disease-like spots in the rice mutant *noe1* were mainly due to the accumulation of hydrogen peroxide in the plant, which activated nitrate reductase, resulting in a large accumulation of nitric oxide in the body [26]. *SPL33* encodes a eukaryotic translation elongation factor eEF1A consisting of 655 amino acids. The single-base mutation of mutant *spl33* results in premature termination of translation, function loss of the encoded protein, and activation of defense responses. Accompanied by hydrogen peroxide, callose accumulates while increasing resistance to *M. oryzae* and *Xoo* [27].

### 3.6. Abiotic Factors

Some abiotic factors can also cause disease-like spots in rice. When rice is exposed to abnormal light, temperature, etc., an immune response occurs, and PCD, ROS, and immune defense systems are activated, resulting in the occurrence of disease-like spots. The mutant *lmps1* has a lesion mimic phenotype under light, and no lesion mimic lesions appear in the dark [6]. Wang et al. [28] found that high temperatures can lead to a reduction in the number of lesions in the disease-like mutant, but in the late stage of rice growth, the inhibitory effect of temperature on the disease-like phenotype will not be obvious. Mutations in the *OsLSD1* gene can cause a disease-like phenotype in rice. The phenotype of the mutant is affected by temperature. Low temperature conditions before the heading stage can lead to the appearance of disease-like spots, while high temperatures at the three-leaf stage lead to a decrease in the number of leaf spots [29]. High temperature conditions above 30 °C resulted in a large number of necrotic spots in rice *spl7* mutant leaves, but the number of spots decreased significantly when the temperature was lower than 26 °C [30].

## 4. Cloning of Rice Lesion Mimic Gene

The analysis of the reported lesion mimic mutants found that most mutants were of single-gene recessive inheritance, and a few were single-gene dominant and semi-dominant inheritance, all of which followed Mendel’s law of inheritance [1,27]. So far, at least 30 disease-like genes have been cloned in rice (Table 1). The proteins encoded by these plaque-like genes are structurally diverse and functionally distinct, covering every aspect of the life process. In general, the proteins encoded by these disease-like genes can be divided into three categories; the first category includes proteins involved in the SA signaling pathway, CC-NB-LRR proteins of plant innate immunity, membrane-associated proteins, ion channel proteins, clathrin-related proteins, eukaryotic translation elongation factors, and RNA splicing factors. The second category is enzymes, including catalase, oxidoreductase, lipid kinase, protein kinase, cytochrome P450 monooxygenase, acyl transferases, mitosis-related kinases, and E3 ubiquitin-associated enzymes. The third category is lipids, fatty acids, porphyrins, and phenolic compounds. The diversification of plaque-like gene expression products implicates the complex mechanism of plaque-like formation. 

*SPL7* is the first disease-like gene cloned by map-based cloning in rice, located on chromosome 5. *SPL7* encodes a heat stress transcription factor, and the SPL7 protein contains a DNA-binding domain and has a conserved tryptophan. In the mutant, SPL7 undergoes a single-base substitution in the DBD domain (tryptophan→cysteine), resulting in a change in the hydrophobicity of the target protein domain, resulting in loss of protein function, and ultimately a lesion mimic phenotype. From tillering to heading, *spl7* mutants spontaneously formed reddish-brown necrotic spots on the entire leaf surface, and their lesion mimic phenotype was induced by temperature and UV light [30].

*SPL11* is located on rice chromosome 7 and cloned by map-based cloning. The protein encoded by *SPL11* has E3 ubiquitin ligase activity and contains a U-box/armadillo repeat domain. In yeast, proteins with U-box domains can participate in the ubiquitin degradation pathway, and in mammals, proteins with armadillo domains are associated with protein-to-protein interactions. The *spl11* mutant started to present rust-colored necrotic spots on the leaves at the late tiller stage, and the necrotic spots were distributed over the entire leaf and continued until maturity. The *SPL11* gene negatively regulates plant cell death and defense responses through the in vivo ubiquitin system, and the ubiquitin ligase activity of the SPL11 protein depends on the U-box domain. In mutant *spl11*, a single-base mutation results in premature termination of protein translation [24].

*SPL3*, also known as *OsACDR1* or *OsEDR1*, encodes a raf-like mitogen-activated protein kinase with kinase activity and autophosphorylation. The leaves of rice *SPL3*-overexpressing plants spontaneously presented disease-like spots, up-regulated expression of defense response-related genes in vivo, accumulated a large number of phytoalexins and phenolic compounds, and enhanced their resistance to *M. oryzae*. Plants with RNAi of *OsACDR1* had reduced resistance to *M. oryzae*, and the expression of genes related to defense response was down-regulated. Thus, *OsACDR1* positively regulates defense responses in rice [22].

The mutant *ttm1* was identified from a population of insertion mutants of the japonica cultivar Nipponbare retrotransposon *Tos17*. *Tos17* was inserted into the third exon of the *OsPti1a* gene using Tail-PCR technique. The *OsPti1a* gene encodes a plasma membrane protein kinase. The mutant ttm1 homozygous plants presented necrotic spots on the leaf surfaces 30 days after sowing in paddy fields or 40 to 50 days after sowing in greenhouses, the plants were dwarfed, and the mutant phenotype was affected by external conditions. The resistance of *OsPti1a*-overexpressing plants to *M. oryzae* and *Xoo* was reduced, suggesting that OsPti1a protein negatively regulates the defense response mechanism induced by rice disease resistance genes [31,32].

*SPL28* is located on chromosome 1 and cloned by map-based cloning. *SPL28* encodes a clathrin-associated adaptor protein complex 1, medium subunit μ1, AP1M1. In mutant *spl28*, a single base mutation in exon 10 of the *SPL28* gene caused premature termination of translation and resulted in a lesion mimic phenotype. SPL28 protein is localized on the Golgi apparatus and is involved in material transport. The *SPL28* gene can restore the growth of yeast membrane transport mutant apm1-1Δ, and the loss of *SPL28* gene function hinders rice vesicle transport, resulting in necrotic plaques and premature aging. In the process of disease-like spot generation, a large amount of reactive oxygen species, phytochemicals, callose, and phenolic complexes will accumulate in the disease spots and their surrounding cells, which can significantly enhance the resistance to rice blast and bacterial blight [33].

The mutant *nls1* (*necrotic leaf sheath 1*) is a semi-dominant mutant. During plant growth and development, the mutant leaf sheath will spontaneously form HR-like necrotic plaques. The *NLS1* gene is located on chromosome 11 and encodes a CC-NB-LRR protein. In the mutant, the *NLS1* gene has a single-base mutation leading to loss of function of the gene, excess hydrogen peroxide and salicylic acid accumulated in the *nls1* mutant, the expression of defense response-related genes becoming up-regulated, and the resistance to bacterial diseases becoming enhanced. Inhibition of SA-related genes or NPR1 expression did not prevent mutant *nls1* phenotype and activation of defense responses [16].

The mutant *lms* was obtained by EMS mutagenesis of the *japonica* rice variety Hitomebore. The leaves of the *lms* mutant presented reddish-brown necrotic spots from the seedling stage. After flowering, the necrotic spots covered the entire leaf, accompanied by a premature senescence phenotype. The *OsLMS* gene is located on chromosome 2 and encodes a protein containing two double-stranded RNA binding motifs and a carboxyl-terminal domain phosphatase domain. The lesion mimic phenotype is caused by a single base mutation in the *OsLMS* gene, resulting in mRNA splicing errors. Compared with the wild type, the mutant had improved resistance to *M. oryzae* but was more sensitive to chilling injury, suggesting that the *OsLMS* gene negatively regulates abiotic stress and cell death in rice [34].

The *spl29* mutant was derived from the tissue culture progeny of the *japonica* cultivar Zhonghua 11. The gene was cloned by map-based cloning and located on chromosome 8, encoding UDP-N-acetylglucosamine pyrophosphorylase 1 (UAP1). A single base substitution in the *spl29* mutant results in inactivation of the UAP1 enzyme. From the seedling stage to the mature stage, black-brown necrotic spots will spontaneously appear on the leaves. Compared with the wild type, the plant height, ear length, seed setting rate, total number of grains, and thousand-grain weight of the mutant were decreased. The mutants exhibited a premature senescence phenotype with reduced photosynthesis efficiency, chloroplast degradation, up-regulation of senescence-related gene expression and down-regulation of photosynthesis-related gene expression, and increased content of abscisic acid and jasmonic acid. *spl29* accumulates a large amount of reactive oxygen species including O_2_^−^ and H_2_O_2_ in vivo, which increases the resistance to bacterial blight [35].

The *LMR* is located on chromosome 6 and obtained by map-based cloning. *LMR* encodes an AAA (various cellular activities) type ATPase. From the seedling stage, reddish-brown necrotic spots appeared on the leaves of the mutant *lmr* until the mature stage. The lesion-like phenotype is due to the premature termination of translation caused by a single base mutation in the *LMR* gene. In the mutants, H_2_O_2_ is excessively accumulated, the expression of disease-related genes *PBZ1* and *PR1* is up-regulated, and the resistance to rice blast and bacterial blight is enhanced, so the *LMR* gene negatively regulates the defense response and cell death of rice [36].

The mutant *oscul3a* is derived from the EMS-mutated mutant library of Zhonghua 11, which can significantly improve the resistance to rice blast and bacterial blight. *Os CUL3a* is located on rice chromosome 2 and encodes an OsCUL3a protein with a Cullin domain. Premature termination of OsCUL3a protein translation in mutants results in cell death and a broad-spectrum disease-resistant phenotype. OsCUL3a can interact with OsRBXs to form a Cullin-Ring complex subunit E3 ubiquitin ligase in vivo, and negatively regulate PCD and immune stress responses in rice by targeting the degradation of OsNPR1 protein [25].

In the spotted leaf mutant *spl35*, small reddish-brown lesions appeared from the tip to the bottom of the leaf from the three-leaf stage, and these spots gradually enlarged and spread on each fully expanded leaf. Chlorophyll a and b and carotenoid contents of *spl35* were significantly reduced, H_2_O_2_ accumulated, chloroplast development was abnormal, but resistance to rice blast and bacterial blight was enhanced. The *SPL35* gene encodes a CUE domain-containing protein that is constitutively expressed, and its excessive or disturbed expression can induce lesions. There is a physical interaction between SPL35 protein and plant ubiquitin-conjugating enzyme protein OsUBC5a in addition to exosome subunit delta proteins Delta-COP1 and Delta-COP2, suggesting that *SPL35* may regulate plant cell death and cell death by participating in ubiquitination and vesicle transport pathways and defensive response [10].

Using ethyl methanesulphonate (EMS) mutagenesis and map-based cloning, we identified an *Oryza sativa ACL-A2* mutant allele, termed *spotted leaf 30-1* (*spl30-1*), in which an A-to-T transversion converted an Asn at position 343 to a Tyr (N343Y), causing a recessive mutation that led to a lesion mimic phenotype. Compared with wild-type plants, *spl30-1* significantly reduced ACL enzymatic activity, accumulated high reactive oxygen species, and increased the rate of nuclear DNA degradation. CRISPR/Cas9-mediated insertion/deletion mutation analysis and complementation analysis confirmed that the phenotype of *spl30-1* is caused by the functional defect of OsACL-A2 protein [37].

The lesion mutant gene *ELL1* is closely related to the development and function of chloroplasts, and chloroplast-related genes or proteins are severely affected at both the transcriptional and protein levels. Similarly, a large amount of H_2_O_2_ accumulation and necrotic cells are found in the *ell1* mutant. Severe DNA degradation and aberrant PCD occurred in *ell1* mutants, suggesting that excess ROS accumulation induces DNA damage and ROS-mediated cell death. Mutation of *ELL1* disrupts chloroplast structure, and chloroplast defects lead to ROS accumulation that ultimately triggers PCD, and *ELL1* plays an important role in ROS-mediated cell death [38].

The researchers identified a natural leaf blight mutant *nbl3* from the T-DNA insertion mutant library, which presented spontaneous cell death, ROS accumulation, enhanced disease resistance, salt tolerance, and premature aging, and activated the expression of multiple disease resistance and salt tolerance-related genes. *OsNBL3* encodes a mitochondrial localized pentapeptide repeat (PPR) protein, which is mainly involved in the splicing of intron 4 of mitochondrial gene *nad5*. The deletion of *OsNBL3* leads to the decrease in the activity of mitochondrial respiratory chain complex I in nbl3, the activation of alternative respiratory pathways and the destruction of mitochondrial morphology. Meanwhile, the RNA interference strain of *OsNBL3* presented enhanced disease resistance and salt tolerance similar to *nbl3* [39].

Our research team isolated independent mutant lines of the *OsSCYL2* gene in rice by combining traditional map-based cloning and CRISPR-Cas9-mediated gene editing methods. Mutation of rice *OsSCYL2* produces a novel phenotype—light-dependent hypersensitive response-like (HR) cell death. SCY1-LIKE2 (SCYL2) is an evolutionarily conserved protein kinase in eukaryotes. Rice *scyl2* mutants presented only minor growth defects, and the most obvious change was reddish-brown necrotic spots on new leaves since tillering, and the formation of lesions was light-dependent. Furthermore, *OsSCYL2* mutations can lead to cell death and accumulation of reactive oxygen species. Further detailed phenotypic analysis revealed that although *scyl2* mutant lines exhibited premature senescence and defects in the photosynthetic system, enhanced resistance to causative pathogens was observed [40].

**Table 1:** Partial of the cloned genes of rice lesion mimic mutants.

Name	Gene ID	Gene Function	References
*SPL7*	*LOC_Os05g45410*	heat shock transcription factor	[30]
*SPL11*	*LOC_Os12g38210*	U-box E3 ubiquitin ligase	[24]
*OsNPR1*	*LOC_Os01g09800*	disease resistance factor	[41]
*OsLSD1*	*LOC_Os08g06280*	zinc finger protein	[29]
*SPL18*	*LOC_Os10g11980*	acyltransferase	[42]
*OsPti1a*	*LOC_Os05g04520*	receptor-like protein kinase	[31]
*OsSSI2*	*LOC_Os01g69080*	fatty acid desaturase	[18]
*OsACDR1*	*LOC_Os03g06410*	mitogen kinase kinase kinase	[22]
*OsSL*	*LOC_Os12g16720*	P450 monooxygenase	[43]
*SPL28*	*LOC_Os01g50770*	grid-associated adaptor protein complex	[33]
*GF14e*	*LOC_Os02g36974*	14-3-3 protein	[23]
*RLIN1*	*LOC_Os04g52130*	coproporphyrinogen III oxidase	[20]
*NLS1*	*LOC_Os11g14380*	CC-NB-LRR protein	[16]
*RLS1*	*LOC_Os02g10900*	Novel protein containing NB-ARM domain	[44]
*OsCATC*	*LOC_Os03g03910*	catalase C	[26]
*OsHPL3*	*LOC_Os02g02000*	Lipid hydroperoxide lyase	[45]
*OsLMS*	*LOC_Os02g42600*	RNA binding protein	[34]
*OsCs1F6*	*LOC_Os08g06380*	cellulose synthase	[46]
*OsNPR1*	*LOC_Os01g09800*	transcriptional coactivator	[47]
*FGL*	*LOC_Os10g35370*	protochlorophyllate oxidoreductase B	[21]
*LMR*	*LOC_Os06g03940*	AAA-type ATPase	[36]
*SPL5*	*LOC_Os02g08070*	SF3b3-type splicing factor	[48]
*SPL29*	*LOC_Os08g10600*	UAP1	[35]
*OsWAK25*	*LOC_Os03g12470*	wall-associated receptor-like kinase 25	[49]
*LLB*	*LOC_Os07g14350*	leucine carboxyl methyltransferase	[50]
*OsPLS1*	*LOC_Os06g45120*	vacuolar-type H+-ATPase subunit A1	[51]
*EBR1*	*LOC_Os05g19970*	Ring-type E3 ubiquitin ligase	[52]
*OsCUL3a*	*LOC_Os02g51180*	cullin-RING-like ubiquitin ligase complex	[25]
*SPL32*	*LOC_Os07g46460*	Fd-GOGAT	[53]
*SPL33*	*LOC_Os01g02720*	translation elongation factor	[27]
*SDS2*	*LOC_Os01g57480*	S-domain receptor-like kinase	[54]
*OsPELOTA*	*LOC_Os04g56480*	eukaryotic translation release factor	[55]
*SPL35*	*LOC_Os03g10750*	CUEDC protein	[10]
*SPL30*	*LOC_Os12g37870*	ATP-citrate lyase A2 subunit	[37]
*OsJAZ13*	*LOC_Os10g25230*	jasmonate ZIM-domain protein	[56]
*ELL1*	*LOC_Os12g16720*	Cytochrome P450 Monooxygenase	[38]
*OsNBL3*	*LOC_Os03g06370*	pentatricopeptide repeat protein	[39]
*OsSCYL2*	*LOC_Os01g42950*	A conserved clathrin-coated vesicle component	[40]

## 5. Disease Resistance of Lesion Mimic Mutants

In the long-term evolution process, plants themselves have formed a complex and effective defense mechanism against the invasion of various external pathogens. The first barrier of a plant is its own epidermis, cuticle, waxy layer, and cell wall. When the first barrier of a plant cannot stop the invasion of pathogenic bacteria; the plant body will activate the immune system to resist further infection by pathogenic bacteria. The plant immune system is divided into two aspects: the first aspect is the immune response (PAMP-triggered immunity, PTI) induced by pathogen-associated molecular patterns (PAMPs), which is the pattern recognition receptor protein of the plant cell membrane; The second aspect is the immune response (effector-triggered immunity, ETI) induced by a pathogenic bacteria effector. Some pathogens can secrete a variety of effectors, thereby interfering with the plant’s PTI response. After plant sensing, molecular receptors (such as R gene) of the body recognize the effector of pathogenic bacteria, thereby triggering the second layer of defense response ETI [57]. ETI is accelerated and amplified PTI. The occurrence of ETI can lead to enhanced plant resistance, usually with hypersensitivity reactions at the site of pathogen infection, causing cell death.

**Figure 2:**
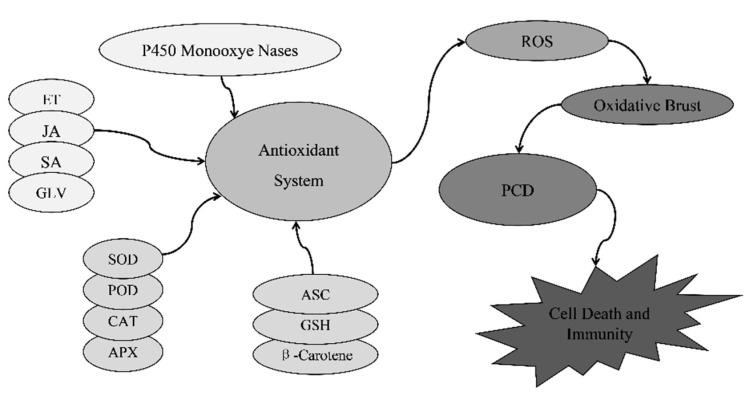
Mechanism of PCD in some rice lesion-mimicking mutations. ET: Ethylene; GLV: Green leaf volatiles; JA: Jasmonic acid; SA: Salicylic acid; SOD: Superoxide dismutase; CAT: Catalase; POD: Peroxidase; APX: Aseorbateperoxidase; ASC: Ascorbic acid; GSH: Glutathione; ROS: Reactive oxygen species; PCD: Programmed cell death.

When the lesion mimic mutants make immune defense responses, they will be accompanied by changes in a variety of physiological indicators at the cellular level, such as reactive oxygen species, phytoalexins, phenolic compounds, and calluses. The improvement of these physiological indicators will induce the hypersensitive response (HR) of plants and effectively resist the invasion of pathogenic bacteria, thereby presenting increased resistance of the plant [40,42,58,59,60]. Studies have indicated that most lesion-mimicking mutants have better resistance to pathogens than wild-types. For example, after mutation of the rice disease-like gene *SPL28*, phytopoxin and callose are accumulated in the mutant, and the mutants indicated higher resistance to rice blast and bacterial blight [33]; mutants such as *spl29*, *spl35*, and *scyl2* had improved bacterial blight resistance [10,35,40]. Reactive oxygen free radicals and their intermediate metabolites are the first type of signaling molecules to be discovered. The production of most lesions makes the production rate of intracellular reactive oxygen species faster than the decomposition rate, resulting in the accumulation of reactive oxygen species, thereby activating intracellular antioxidants. The enzyme system maintains the balance system of intracellular reactive oxygen species, and when the reactive oxygen species reaches a certain level, the PCD pathway in the cell will be activated, and finally, disease-like spots will appear on the leaves. Ethylene, green leaf volatiles, jasmonic acid, and salicylic acid are also involved in plant disease resistance and stress responses during PCD in rice [61,62] (Figure 2).

## 6. Discussion and Perspective

Rice is a major food crop and a model plant for functional genomics research. Exploring rice disease spots and disease resistance mechanisms is one of the current research hotspots. The formation process of rice disease-like spots is affected by many factors, and rice resistance is also related to the disease-like process. In the process of disease-like disease, lesion-mimicking mutants usually induces an immune response in the plant, thereby inducing the defense system, so that it will indicate a certain resistance to rice diseases, and it also affects agronomic traits such as plant height, effective panicle number, grain number per panicle, and seed-setting rate. Therefore, the cloning and functional analysis of rice disease-like mutant genes become increasingly important, and the molecular regulation mechanism of the disease-like disease needs to be further studied.

Lesion-mimicking mutants are ideal materials for studying the mechanisms of programmed cell death and defense response formation in plant cells. Most of the cloned lesion mimic mutant genes indicated good resistance to rice blast and bacterial blight. Over the past 100 years, breeders have used “disease-resistance genes” to improve crop resistance and put forward the “gene-for-gene” theory of disease resistance. In recent years, the use of molecular biology methods has made people have a more in-depth understanding of the molecular mechanism of plant disease resistance, but there are still some problems in the application of disease resistance breeding, such as the resistance of varieties being singular and easily lost, and recessive disease resistance genes are not easy to use. Therefore, we need to continuously explore and utilize high-quality rice germplasm resources, aggregate the main disease resistance functional genes, play a synergistic disease resistance role, enhance their broad-spectrum and long-term disease resistance, and cultivate new rice varieties with disease resistance.

## Data Availability

All of the data generated or analyzed during this study are included in this published article.
